# Supplementary material for: Estimating the burden of the COVID-19 pandemic on mortality, life expectancy and lifespan inequality in England and Wales: a population-level analysis
Source: J Epidemiol Community Health. 2021 Jan 19:jech-2020-215505. doi: 10.1136/jech-2020-215505 (PMC7818788; doi:10.1136/jech-2020-215505)

Title: Estimating the burden of COVID-19 on mortality, life expectancy and lifespan inequality in England and Wales: A population-level analysis

Authors: José Manuel Aburto, Newton fellow[1,2], Ridhi Kashyap, associate professor[1], Jonas Schöley, postdoctoral fellow[2], Colin Angus, senior research fellow[3], John Ermisch, professor[1], Melinda C. Mills, professor[1], Jennifer Beam Dowd, associate professor[1]

Affiliations:
[1] Leverhulme Centre for Demographic Science, Department of Sociology and Nuffield College, University of Oxford, 42-43 Park End Street, OX1 1JD Oxford, UK.

[2] Interdisciplinary Centre on Population Dynamics, University of Southern Denmark, Odense 5000, Denmark.

[3] School of Health and Related Research, University of Sheffield, Regent Court, Regent Street, S1 4DA Sheffield, UK

Correspondence to:
José Manuel Aburto
42-43 Park End Street, OX1 1JD Oxford, UK.
Email: jose-manuel.aburto@sociology.ox.ac.uk
Tel: +45 31712122
ORCID: 0000-0002-2926-6879

OR

Ridhi Kashyap
Nuffield College, New Road, Oxford OX1 1NF
Email: ridhi.kashyap@sociology.ox.ac.uk
ORCID: 0000-0003-0615-2868

Section 1. Estimation of the baseline mortality risk using 4 different approaches using training data from 2010 to week 10 of 2020 by age and sex.

1. Generalized Additive Model regression on weekly death counts assuming a Negative Binomial distribution to account for overdispersion of deaths during the period we study[1]. The model includes
   a. log-linear long-term mortality trends stratified by age group and sex,
   b. smoothed effects for mortality over age and seasonality stratified by sex,
   c. a smoothed interaction for mortality between age and seasonality stratified by sex, and
   d. age and sex specific effects for "special weeks", i.e. the first and last week of a year and week 21 where holidays cause registration delays,
   e. logged exposure times as offset.

   The structure of the model is as follows:

$$log\left(E(Y_i)\right) = \beta_0 + \beta_1 sex_i + \beta_2 agegroup_i + \beta_3 specialweek_i + \beta_4 time_i + \beta_5 sex_i * agegroup_i + \beta_6 time_i * sex_i + \beta_7 time_i * agegroup_i + \beta_8 time_i * sex_i * agegroup_i + \beta_9 specialweek_i * sex_i + \beta_{10} specialweek_i * agegroup_i + \beta_{11} specialweek_i * sex_i * agegroup_i + f_1(age_i | sex_i) + f_2(weekofyear_i|sex_i) + f_3(weekofyear_i, age_i|sex_i) + log(\theta_i)$$

   Where $E(Y_i)$ are the expected deaths in a given week and population stratum, θ_i are the population exposures, and f are smoothed functions of continuous covariates. f_1 is a penalized spline for the age effect with a dimension 6 basis, $f_2$is a penalized cyclic spline over week of year for the seasonality effect with a dimension 8 basis and $f_3$is a smoothed interaction of age and seasonality.

2. The second approach is a Generalized Additive Model assuming a Poisson distribution with the same structure as above.

3. The third approach is a Generalized Linear Model regression on weekly deaths assuming a Poisson distribution and featuring trigonometric terms for the seasonal effect. These so-called Serfling models [2,3] are used to estimate baseline mortality during influenza epidemics. The basic structure of the model is as follows:

$$log\left(E(Y_i)\right) = \beta_0 + \beta_1 time_i + \gamma_2 sin\left(\frac{2\pi\ weekofyear_i}{52}\right) + \gamma_3 cos\left(\frac{2\pi\ weekofyear_i}{52}\right) + \gamma_4 sin\left(\frac{2\pi\ weekofyear_i}{26}\right) + \gamma_5 cos\left(\frac{2\pi\ weekofyear_i}{26}\right) + log(\theta_i),$$

   where all the terms are fully interacted with age and sex and "special weeks" are included in the same way as in the Generalized Additive models.

4. We constructed an empirical baseline mortality based on the average mortality rate over the previous five years 2015-19 within each week and stratum. The associated deaths from this approach result from multiplying the average death rates by the population exposed to the risk.

Aburto JM, *et al. J Epidemiol Community Health* 2021;0:1–6. doi: 10.1136/jech-2020-215505

5. **Excess deaths produced with different models.**

Table 1. Total excess deaths by the end of week 47 estimated with 4 different models with 95% prediction intervals in England and Wales.

	Female			Male			Total		
**Model**	**Excess**	**.05 PI**	**.95 PI**	**Excess**	**.05 PI**	**.95 PI**	**Excess**	**.05 PI**	**.95 PI**
GAM Negative Binomial	25,629	23,244	27,954	31,791	29,539	33,853	57,419	54,197	60,752
GAM Poisson	25,597	24,721	26,487	31,724	30,893	32,667	57,321	56,044	58,538
GLM Poisson (Serfling)	25,341	24,464	26,188	31,463	30,605	32,337	56,804	55,605	57,933
Average mortality	22,087	21,269	22,969	26,969	26,110	27,801	49,056	47,867	50,319

Table 2. Total excess deaths by the end of week 47 estimated with 4 different models by age and sex with 95% predictive intervals in England and Wales.

		Female			Male		
**Model**	**Age group**	**Excess**	**.05 PI**	**.95 PI**	**Excess**	**.05 PI**	**.95 PI**
GAM Negative Binomial	0	-72	-142	-3	-88	-161	-14
	15	324	172	467	329	109	527
	45	2,584	2,135	3,044	5,275	4,634	5,907
	65	3,440	2,845	4,070	6,395	5,531	7,221
	75	7,760	6,636	8,903	10,831	9,365	12,223
	85	11,592	9,536	13,674	9,049	7,704	10,338
GAM Poisson	0	-76	-139	-12	-90	-164	-15
	15	328	201	452	334	166	507
	45	2,586	2,337	2,830	5,269	4,971	5,565
	65	3,407	3,110	3,735	6,283	5,923	6,662
	75	7,719	7,269	8,181	10,770	10,296	11,235
	85	11,634	11,063	12,264	9,159	8,684	9,624
GLM Poisson (Serfling)	0	-75	-139	-8	-89	-160	-18
	15	322	196	441	331	162	491
	45	2,583	2,313	2,841	5,248	4,939	5,581
	65	3,332	3,029	3,661	6,225	5,861	6,597
	75	7,679	7,242	8,120	10,665	10,208	11,142
	85	11,500	10,916	12,081	9,083	8,606	9,535
Average mortality	0	-187	-253	-122	-234	-308	-156
	15	252	137	369	176	10	343
	45	2,146	1,863	2,418	4,644	4,324	4,976
	65	2,767	2,459	3,083	5,120	4,761	5,480
	75	5,676	5,233	6,103	8,495	8,008	8,962
	85	11,433	10,843	12,066	8,768	8,275	9,256

References

1 Wood SN. *Generalized Additive Models: An Introduction with R, Second Edition*. CRC Press 2017.

2 Nielsen J, Krause TG, Mølbak K. Influenza-associated mortality determined from all-cause mortality, Denmark 2010/11-2016/17: The FluMOMO model. *Influenza Other Respir Viruses* 2018;**12**:591–604. doi:10.1111/irv.12564

3 Serfling RE. Methods for Current Statistical Analysis of Excess Pneumonia-Influenza Deaths. *Public Health Rep 1896-1970* 1963;**78**:494–506. doi:10.2307/4591848

Figure 1. Expected (lines) vs. observed deaths (points) counts based on the 4 approaches described above for males by age groups (rows) 0-14, 15-44, 45-64, 65-74, 75-84 and 85-older years of age. Shaded areas indicate 95% prediction intervals.

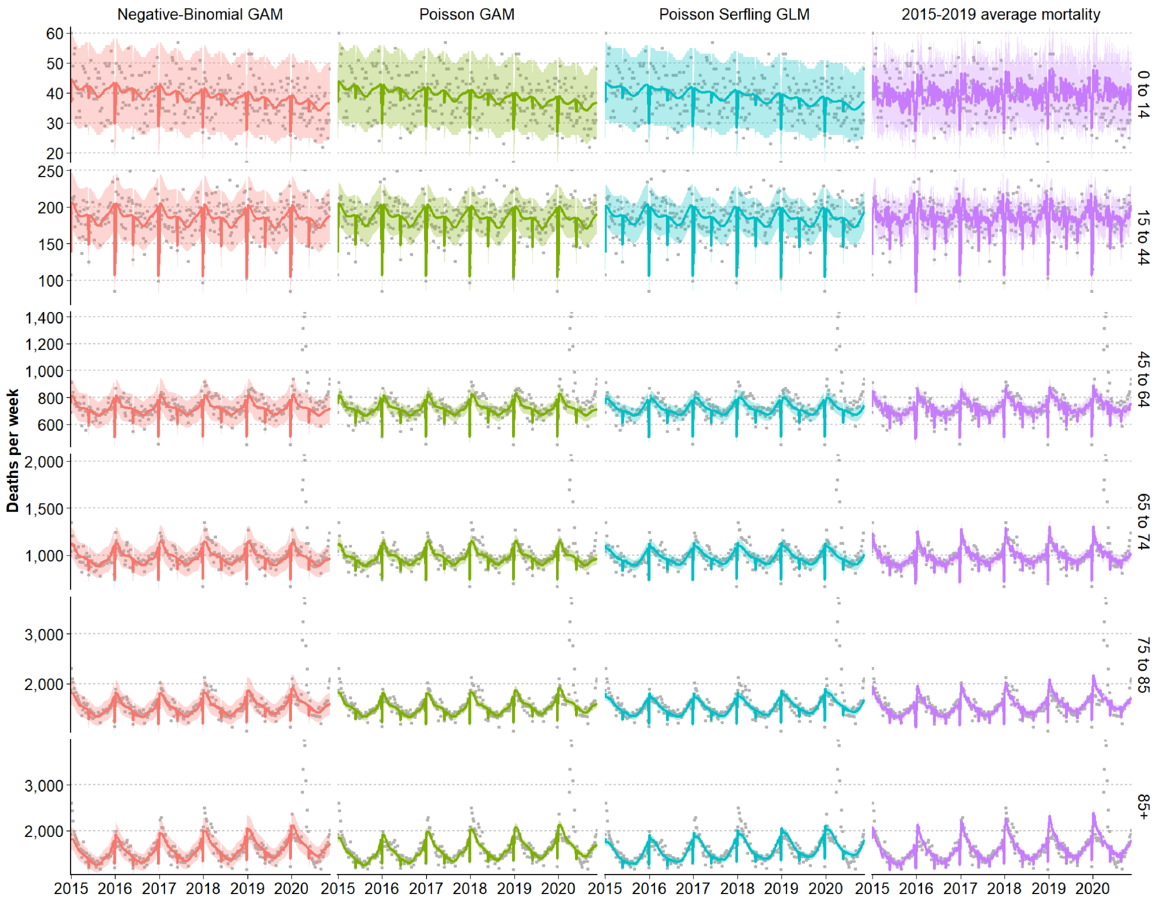

Figure 2. Expected (lines) vs. observed deaths (points) counts based on the 4 approaches described above for females by age groups (rows) 0-14, 15-44, 45-64, 65-74, 75-84 and 85-older years of age. Shaded areas indicate 95% prediction intervals.

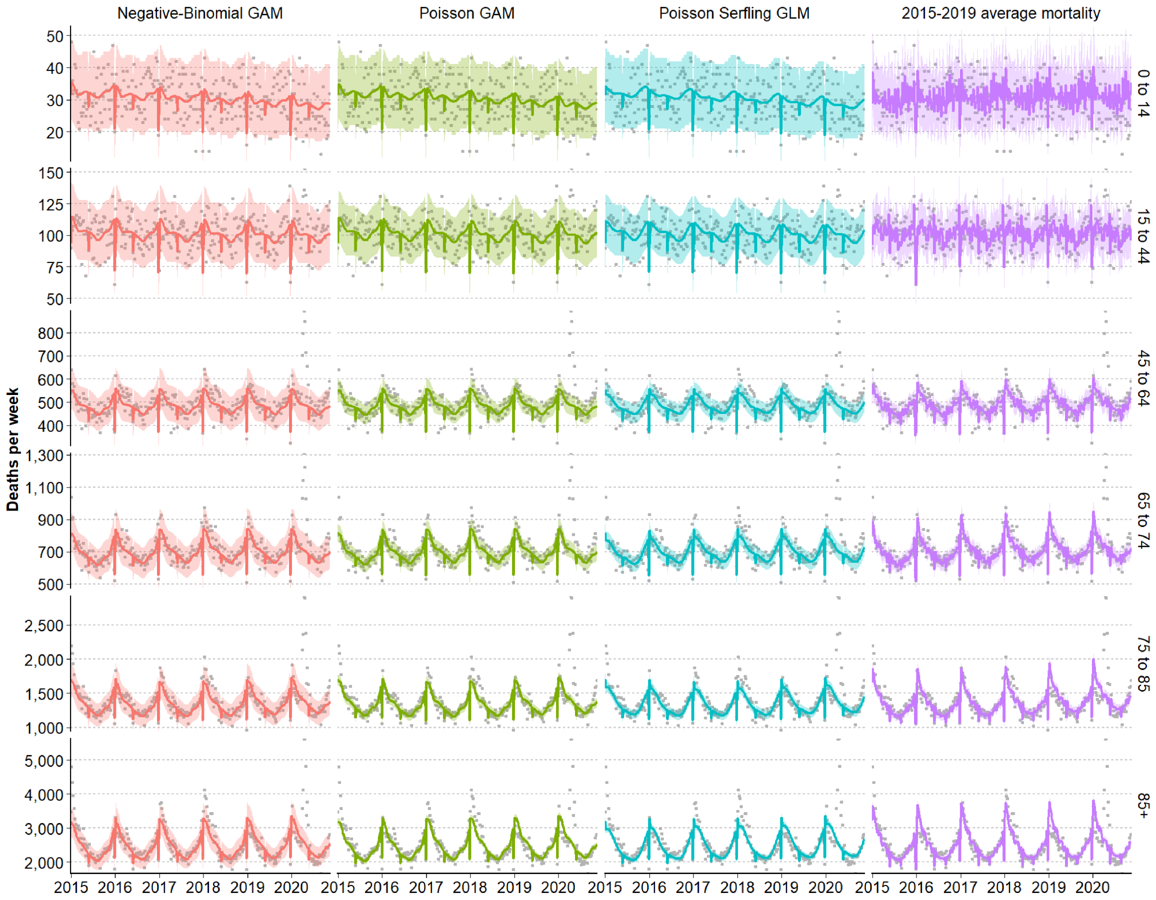

Figure 3. Sex ratio males/females of death rates during the course of the pandemic by age groups (rows) 0-14, 15-44, 45-64, 65-74, 75-84 and 85-older years of age.

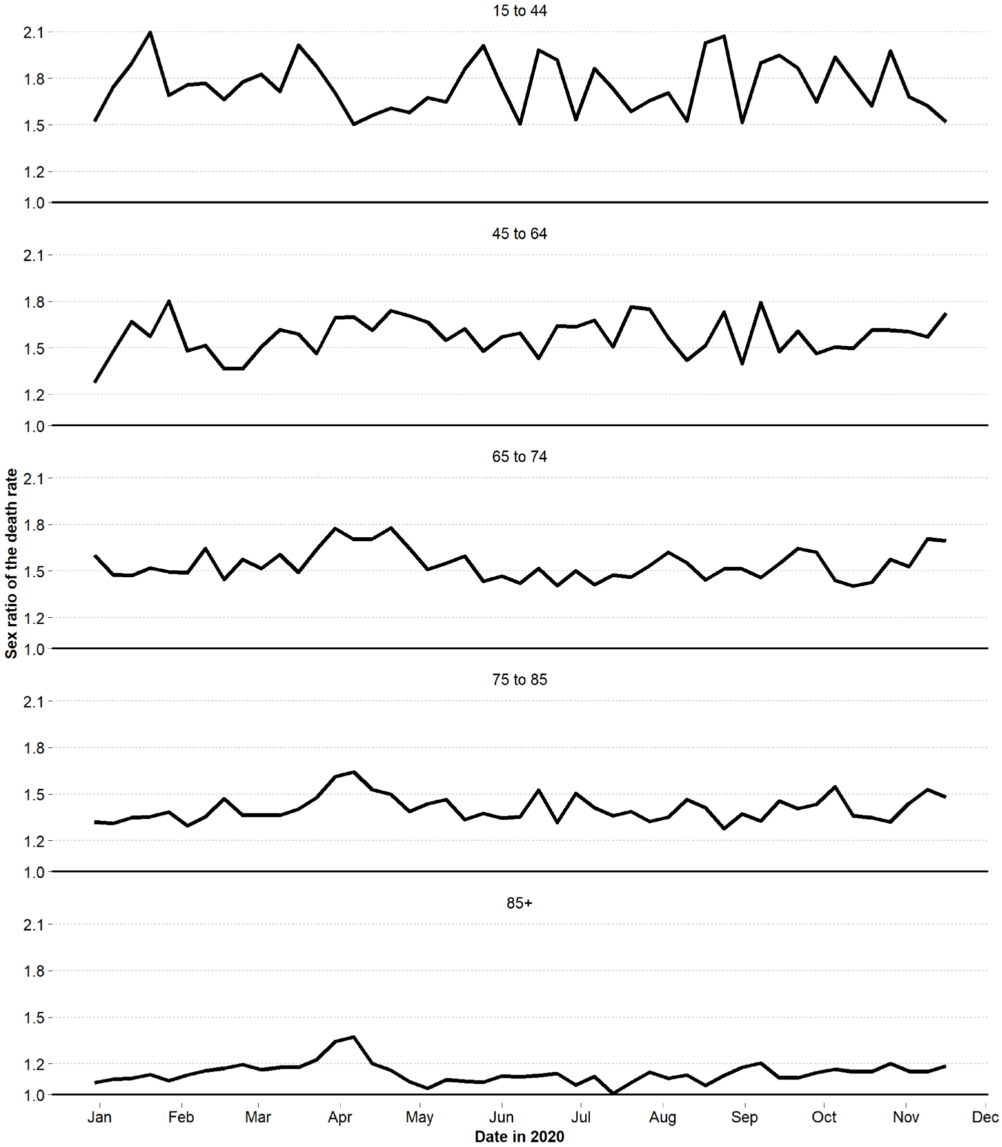

Supplement: Supplementary file 1 [file jech-2020-215505supp001.pdf]
